# Ovine *LncRSFD1* Mined from RNA-Seq: Identification, Expression Profile, Promotion of Preadipocyte Differentiation, Promoter Activity, and Its Polymorphisms Related to Phenotypic Traits

**DOI:** 10.3390/ani14243631

**Published:** 2024-12-16

**Authors:** Hongwei Xu, Yunyun Luo, Mengyang Zhang, Chuanying Pan, Xianyong Lan, Juanshan Zheng

**Affiliations:** 1 College of Life Science and Engineering, Northwest Minzu University, Lanzhou 730030, China; xuhongwei@xbmu.edu.cn; 2 College of Animal Science and Technology, Northwest A&F University, Yangling 712100, China; lyy980909@126.com (Y.L.); zhangmengyang0518@163.com (M.Z.); panyu1980@126.com (C.P.)

**Keywords:** sheep, tail fat, *lncRSFD1*, preadipocytes, single nucleotide polymorphism (SNP), promoter

## Abstract

Tail fat is essential for sheep survival in extreme environments, yet its significance is often overlooked, leading to the decline of fat-tailed breeds. This study identified a novel lncRNA, *lncRSFD1* (*TCONS_00054953*), through transcriptome sequencing, showing differential expression in the tail adipose tissues of Lanzhou Fat-Tailed (LFT) sheep and Tibetan (TS) sheep. Highly expressed in adipose tissues, *lncRSFD1* inhibits preadipocyte proliferation and promotes 3T3-L1 differentiation, suggesting its role in regulating fat deposition. Located in both the cytoplasm and nucleus, *lncRSFD1* targets the neighboring gene *PDE4DIP* and may function as a molecular sponge for conserved miRNAs, including oar-miR-30a-3p, *oar-miR-329b-5p*, and *oar-miR-431*, which are known to influence fat and muscle-related physiological processes. Moreover, the core promoter of *lncRSFD1* (−2607 bp to −1776 bp) harbors four SNPs (g.-2429G>A, g.-2030T>C, g.-2016C>T, g.-2015G>A) significantly associated with growth traits such as body height in Guiqian Semi-Fine Wool (GSFW) sheep. These findings suggest *lncRSFD1* plays a key role in fat deposition and growth regulation, offering new insights into the molecular mechanisms of lncRNAs in sheep. It provides a potential target for genetic improvement and molecular breeding to enhance fat deposition and adaptability in sheep breeds.

## Simple Summary

Sheep in extreme environments rely on tail fat for survival, but the importance of this trait is often overlooked, leading to the decline of fat-tailed breeds. This study discovered a novel molecule named *lncRSFD1*, a type of non-coding RNA, which is highly active in sheep fat tissues. It helps control fat deposition by slowing the growth of early fat cells and encouraging their maturation. *lncRSFD1* interacts with neighboring genes and important miRNAs that influence fat and muscle processes. Additionally, specific variations in the genetic region controlling *lncRSFD1* are linked to growth traits like body height in certain sheep breeds. These findings shed light on how genetic factors regulate fat storage and growth in sheep, offering valuable tools for improving sheep breeds through selective breeding. This could enhance fat deposition and adaptability, particularly for sheep in challenging environments, supporting both their survival and agricultural productivity.

## 1. Introduction

Adipose tissue, crucial for energy storage and homeostasis, significantly affects body composition and meat quality in sheep. Some breeds accumulate fat in their tails and hindquarters in addition to subcutaneous, abdominal, and visceral fat. According to “*China Livestock and Poultry Genetic Resources (Sheep Volume)*”, sheep can be categorized into five tail types: short fat-tail, long fat-tail, short thin-tail, long thin-tail, and fat-rump, and approximately 25% of the world’s sheep are fat-tailed, a type known for storing energy in their tails to survive harsh environments [1,2]. However, excessive tail fat deposition reduces fat in primary meat areas, lowering feed efficiency, raising production costs, and decreasing tail fat utilization, leading to a decline in fat-tailed sheep breeds worldwide [3]. Some fat-tailed breeds, valued for their resilience and adaptability, are now endangered [4]. Thus, understanding the molecular mechanisms of tail fat deposition is crucial for developing better breeding practices with desired growth traits.

The biological mechanisms underlying adipocyte proliferation and differentiation are intricate, involving numerous functional genes, transcription factors (TFs), signaling pathways, and others, which work cooperatively to activate adipocyte-specific gene expression, inducing adipocyte phenotype. For instance, TFs such as *peroxisome proliferators-activated receptor γ* (*PPARγ*) and *CCAAT*/*enhancer-binding protein* (*C*/*EBP*) play crucial roles in controlling adipocyte development in vivo and in vitro, and *PPARγ* can induce each other’s expression, promoting and maintaining the mature adipocyte phenotype [5,6,7]. Adipogenesis also involves the regulation of small molecules like vitamins and a plethora of enzymes, including key enzymes regulating fatty acid synthesis like fatty acid synthase and those related to fat breakdown such as monoacylglycerol lipase and hormone-sensitive triglyceride lipase [8,9]. Adipocytes also secrete numerous signaling proteins known as adipokines, including adiponectin. These adipokines play essential roles in energy metabolism, cell growth, and immune regulation in the body [10]. In recent years, long non-coding RNAs (lncRNAs) have been discovered to participate in regulating adipogenesis and fat deposition.

lncRNAs, which are transcripts longer than 200 nucleotides with limited protein-coding potential [11], can interact with nucleic acids and proteins, contributing to chromatin remodeling, splicing regulation, mRNA degradation, and multi-level gene expression control, including before, during, and after transcription of protein-coding genes. Nowadays, advances in genomics technologies have led to the discovery of numerous candidate genes and non-coding RNAs that influence tail fat deposition in sheep [12]. Fei et al. identified 17 differentially expressed miRNAs related to fat metabolism in the tail fat of Hu sheep (short-fat tail) and Tibetan sheep (short-thin tail) using miRNA-Seq [13]. Differentially expressed lncRNAs (de-lncRNAs) in subcutaneous adipose tissue of Dorper sheep (long-fat tail) and Small Tail Han sheep (short-fat tail) were found to target mRNAs, participating in processes related to unsaturated fatty acid biosynthesis, metabolism, and adipocyte differentiation, which are associated with fat deposition [14]. However, the detailed molecular mechanisms governing the regulation of fat development by these candidate functional genes and non-coding RNAs remain to be fully elucidated in the field of genetics. Apart from sheep, *lncMYOZ2* has been shown to be differentially expressed in the back muscle and fat tissues of Duroc pigs (lean-type pigs) and Chinese indigenous fat-type pigs, Meishan pigs [15]. Additionally, in buffalo muscle and fat tissues, a de-lncRNA, *lncSAMM50*, which is highly expressed in fat tissue, promotes adipogenic differentiation of buffalo preadipocytes by upregulating the expression of adipogenic differentiation-related genes [16].

The functional roles of lncRNAs depend on their subcellular localization. Cytoplasmic lncRNAs can modulate mRNA stability, translation, and post-translational modifications [17]. Cytoplasmic lncRNAs can also act as competing endogenous RNAs (ceRNAs) by interacting with miRNAs, thus relieving miRNA-mediated inhibition of protein-coding genes [18]. In contrast, nuclear lncRNAs expressed in the nucleus can directly influence transcription by recruiting transcription factors or epigenetic complexes, thereby inhibiting or activating the expression of downstream genes [19]. Additionally, lncRNAs can regulate nearby genes or distant genes in cis or trans, and cis-lncRNAs may involve regulatory elements in gene promoters, transcription or splicing processes, or RNA sequences/structures [20]. While the molecular mechanisms underlying the regulation of lipid metabolism by lncRNAs and their involvement in adipogenesis have been increasingly studied, much of the research has focused on model organisms like mice or genetically similar pigs. In contrast, research on lncRNAs in sheep, particularly their role in regulating adipogenesis in sheep tail fat, remains limited.

Based on our previous transcriptome data of ovine tail adipose tissue [4], lncRNA 1 (TCONS_00054953, *lncRSFD1*), which is differentially expressed in long fat-tailed and short thin-tailed sheep, was hypothesized to regulate fat deposition in sheep. Different sheep breeds with varying tail types were used to investigate this hypothesis. Lanzhou fat-tailed sheep (LFT) have tail fat comprising about 11.46% of their body weight and are recognized for rapid growth, large body size, high meat quality, and substantial fat production, primarily found in the suburbs of Lanzhou city in Gansu province [21]. Small-tailed Han sheep (STH), representing short fat-tailed sheep, are characterized by prolificacy, early maturation, rapid growth, and strong adaptability [22]. Tibetan sheep (TS), representing short thin-tailed sheep, have less fat deposition in their tails and greater omental and visceral fat deposition [16], while Guiqian semi-fine wool sheep (GSFW), representing long thin-tailed sheep, are known for cold resistance, disease resistance, adaptability, and superior meat and wool production traits [23]. This study not only explored the role of *lncRSFD1* in regulating fat deposition but also screened and identified genetic variants in the core promoter region affecting sheep body size traits. This research aims to elucidate the molecular mechanisms of ncRNA regulation in sheep fat deposition and accelerate the breeding of high-quality meat-producing sheep breeds.

## 2. Materials and Methods

### 2.1. Collection of Sheep Tissue Samples

In this study, ear tissue samples were collected from a total of 402 sheep individuals. Among them, 52 LFT samples were obtained from Ruilin Technology Breeding Co., Ltd. (Yongjing County, Linxia Hui Autonomous Prefecture, Gansu Province, China). The remaining 350 GSFW samples were provided by Xinwumeng Company in Bijie, Guizhou, China, including 66 rams aged 1 year and 284 adult ewes aged 2.5 to 9 years. The experimental sheep were in normal development and good health. Various parameters, including body weight (kg), body height (cm), body length (cm), chest depth (cm), chest width (cm), chest circumference (cm), girth (cm), and withers height (cm), were recorded according to the same measurement standards.

Various tissue samples, including the heart, liver, spleen, lung, kidney, small intestine, longest back muscle, tail fat, subcutaneous fat, greater omentum, and testis, were collected from three LFT and three TS sheep at approximately 6 months old for gene expression analysis, provided by Ruilin Technology Breeding Co., Ltd.

### 2.2. DNA and RNA Extraction and qRT-PCR

Total RNA from various tissues was extracted using the AG RNAex Pro Reagent (Aikerui Biotech, Changsha, China) following the manufacturer’s instructions, while sheep genomic DNA was extracted from ear tissue samples using the phenol-chloroform method. The quality and concentration of DNA and RNA were assessed using agarose gel electrophoresis and NanoDrop (Thermo Fisher Scientific, Waltham, MA, USA).

The RNA reverse transcription followed the instructions of the Evo M-MLV Reverse Transcription Kit II (including gDNA removal for qPCR) (Aikerui Biotech, Changsha, China). qPCR reactions were carried out using the two-step PCR reaction program provided in the ChamQ SYBR qPCR Master Mix (Vazyme, Nanjing, China) instruction manual and performed on a LightCycler 96 instrument (Roche Pharmaceuticals Ltd, Shanghai, China). The 2^−ΔΔCt^ method was used to analyze the mRNA expression levels of *lncRSFD1* and various genes in different tissues. Real-time quantitative PCR (qRT-PCR) primers were designed using the NCBI-Blast website with reference to the sheep *lncRSFD1* sequence, as well as the mRNA sequences of the sheep *PDE4DIP* gene and the housekeeping gene *GAPDH* found in the GenBank database. The primer sequences are provided in Supplemental Tables and were synthesized by Sangon Biotech (Shanghai, China).

### 2.3. Cell Culture, Transfection, and Luciferase Activity Analysis

3T3-L1 cells were cultured in a 7% CO_2_ incubator at 37 °C until they reached approximately 60–70% confluency. Cell transfection experiments were conducted following the Lipofectamine™ 3000 transfection reagent (Thermo Fisher Scientific) manual. After 6 h of transfection, the cells were switched to a basic culture medium (89% DMEM-F12, 10% calf serum, 1% penicillin–streptomycin) and further incubated at 37 °C. For 3T3-L1 preadipocytes that required differentiation induction (kept in the laboratory), when the cell density reached 95%, the basic culture medium was replaced with differentiation-inducing medium.

293T cells were cultured in a complete medium (89% DMEM-F12, 10% fetal bovine serum, 1% penicillin/streptomycin). When the cells reached over 85% confluency, the previous culture medium was discarded, and trypsin was used for a 20-s digestion. Then, the cells were resuspended in complete medium, and seeded into a 96-well plate for transfection when they reached about 70% confluence. The empty plasmid (negative control) and the experimental group plasmid were transiently transfected into the cells 24 h post-transfection, respectively.

After 24 h of cell transfection, the 96-well plate was removed, and the culture medium was discarded. Cell lysis was carried out using 100 μL of Passive Lysis Buffer. Luciferase activity of Firefly Luciferase (F-Luc) and Renilla Luciferase (R-Luc) in each sample was measured using the Dual-Luciferase Reporter Gene Assay Kit (Yeasen, Shanghai, China) under dark conditions, and the ratio of F-Luc to R-Luc was calculated as the relative promoter luciferase activity. Statistical tests and graphical representations were performed using SPSS 25.0 and GraphPad Prism software, respectively.

### 2.4. Cell Proliferation-Related Experiments

The Cell Counting Kit-8 (CCK-8) reagent was obtained from Beyotime Biotechnology (Shanghai, China). Specific experimental procedures were conducted as per the instructions for the Cell Proliferation and Cytotoxicity Assay Kit (E606335). Each sample was prepared with 5 replicates, and 5 μL of CCK-8 Solution reagent was added to each cell well. After incubating at 37 °C in a CO_2_ incubator for 2 h, the absorbance at a wavelength of 450 nm was measured using an enzyme-linked immunosorbent assay (ELISA) reader (with light avoidance).

The EdU staining reagent kit was provided by Bioss Biotechnology Co., Ltd. (Beijing, China). Detailed operational methods followed the instructions for the YF Click-iT EdU Imaging Kit (C6017M). The difference in EdU positivity between different experimental groups, evaluated as the ratio of proliferating cells (red fluorescence) to total cells (blue fluorescence), was used to assess changes in cell proliferation.

### 2.5. Oil Red O Staining

The Oil Red O staining reagent kit was purchased from Solaibao Technology Co., Ltd. (Beijing, China). The experimental procedure was carried out according to the instructions for the Oil Red O Staining Kit (Cell-Specific) (G1262). The specific experimental steps included the following: (1) 5 days after induction of cell differentiation, discard the culture medium, wash the cells with PBS, and fix the cells in 4% paraformaldehyde for 30 min at room temperature; (2) wash with PBS and rinse with 60% isopropanol; (3) discard the 60% isopropanol and add freshly prepared Oil Red O working solution (A solution:B solution = 3:2, filtered) for staining in the dark at room temperature for 20 min; (4) discard the staining solution, rinse with 60% isopropanol for 30 s until the interstitial spaces are clear; (5) wash with PBS and add a sufficient amount of PBS to keep the cells moist while preventing air exposure; (6) observe under a microscope, take bright-field photos, and save. The results of positive fat staining appear in shades of orange to red.

### 2.6. SNP Selection, Genotyping, and Association Analysis

The core promoter region, typically spanning 50–100 base pairs, is a segment of DNA located upstream of a gene that serves as the binding site for the transcription machinery. Based on the identified core promoter sequence of *lncRSFD1* through dual-luciferase reporter assays, SNP detection primers were designed using the online NCBI-BLAST, with forward primer (5′ TGTTTGGGATTGTAAAGAGCCAC 3′) and reverse primer (5′ GTGGCATAGGAACCCTGGAAC 3′), resulting in a product length of 954 bp. Genomic DNA from LFT and GSFW sheep was used as templates for PCR with the SNP detection primers. PCR products were analyzed by agarose gel electrophoresis with a gel concentration of 2%, and the correct PCR products were selected for DNA sequencing analysis (Tsingke Biotech, Xi’an, China). The sequencing results were aligned and analyzed using Chromas 2.1 and BioXM 2.6 software to screen and determine the SNP positions and perform gene genotyping.

Based on the genotype statistics, gene frequencies, allele frequencies, and relevant genetic parameters, including genetic homozygosity (Ho), effective allele numbers (Ne), and polymorphic information content (PIC) (Nei 1973), were calculated. A chi-square goodness-of-fit test was performed to assess whether the experimental sheep population was in Hardy–Weinberg equilibrium (HWE). Linkage disequilibrium (LD) and haplotype analysis of SNPs in the *lncRSFD1* promoter region were conducted using the SHEsis online platform (http://analysis.bio-x.cn/, accessed on 8 October 2023). The D′ value was used to determine whether there was linkage between the sites, with D′ value = 1 indicating complete linkage and D′ value = 0 indicating no linkage.

Using breed and gender as grouping criteria, the SPSS 25.0 statistical software was employed to analyze the associations between individual loci and haplotypes with sheep growth traits in different groups. The Kolmogorov–Smirnov test was used to evaluate the normality of the data. Data comparisons between two groups were conducted using Student’s unpaired *t*-test (for normally distributed data) and non-parametric tests (Mann–Whitney U, for non-normally distributed data). Multiple group data comparisons were performed using tests for homogeneity of variance and one-way analysis of variance. The model used was Yij = μ + Gi + eij, where Yij represents individual phenotype records, μ is the population mean, Gi is the genotype or haplotype effect, and eij is random error. All data are presented as means ± standard error.

### 2.7. Bioinformatics Prediction and Data Analysis

#### 2.7.1. Prediction Analysis of the Biological Characteristics of *lncRSFD1*

The open reading frame (ORF) and protein-coding potential of *lncRSFD1* were predicted using ORF Finder (https://www.ncbi.nlm.nih.gov/orffinder/, accessed on 20 October 2024), Coding Potential Assessment Tool (CPAT) (http://lilab.research.bcm.edu/, accessed on 20 October 2024) [24], and Coding Potential Calculator 2 (CPC2) (http://cpc2.gao-lab.org/, accessed on 20 October 2024) [25]. The secondary structure and cellular molecular localization of *lncRSFD1* were predicted using RNAfold (http://rna.tbi.univie.ac.at/, accessed on 20 October 2024) and lncLocator 1.0 (http://www.csbio.sjtu.edu.cn/bioinf/lncLocator/, accessed on 20 October 2024) [26]. The interaction target of *lncRSFD1* with *PDE4DIP* was predicted and analyzed using the online software IntaRNA 2.0 (http://rna.informatik.uni-freiburg.de/IntaRNA/Input.jsp, accessed on 20 October 2024). Significance tests and data plotting were conducted using SPSS 25.0 and GraphPad Prism 8.0.2 software. Experimental data were presented as “mean ± standard error”.

#### 2.7.2. Prediction of Sheep miRNAs Targeting *lncRSFD1*

Mature miRNA sequences were downloaded from the miRBase 22.0 database (http://www.mirbase.org/, accessed on 20 October 2024). Potential ovine miRNAs targeting *lncRSFD1* were predicted using RNAhybrid (https://bibiserv.cebitec.uni-bielefeld.de/rnahybrid/, accessed on 20 October 2024) and miRanda (https://www.bioinformatics.com.cn, accessed on 20 October 2024) [27]. The intersection of the results from both databases was selected as candidate target genes to reduce false positives in the prediction. Statistical analysis of experimental data was performed using SPSS 25 software.

#### 2.7.3. Bioinformatics Prediction and Construction of Vector

The putative core promoter region of sheep *lncRSFD1* was predicted using online tools BDGP (http://www.fruitfly.org/seq_tools/promoter.html, accessed on 20 October 2024) and Softberry (http://www.softberry.com/berry.phtml?topic=fprom&group=programs&subgroup=promoter, accessed on 20 October 2024) [28]. Transcription factor (TF) binding sites in the core promoter of *lncRSFD1* were predicted using AliBaba 2.1 (http://gene-regulation.com/pub/programs/alibaba2/, accessed on 20 October 2024).

Based on the bioinformatics analysis of the *lncRSFD1* promoter region, a stepwise truncation method was used to design 6 pairs of primers containing restriction enzyme recognition sites for amplifying different deletion fragments of the *lncRSFD1* promoter (Table S1). DNA fragments were amplified using LFT Sheep genomic DNA as a template, followed by gel electrophoresis. The purified DNA fragments were digested with KpnI and XhoI restriction enzymes (Takara, Kyoto, Japan) and ligated into the pGL3-Basic empty vector. After transformation into Trelief^®^ 5α Chemically Competent Cells (QIAGEN, TSC-C01), single clones were selected, and clones were identified by bacterial liquid PCR and sequencing. Subsequently, the plasmids were extracted using a plasmid miniprep kit (Tiangen Bio, Xi’an, China).

## 3. Results

### 3.1. Identification, Molecular Characteristics, and Tissue Expression of lncRSFD1

**Figure 1:**
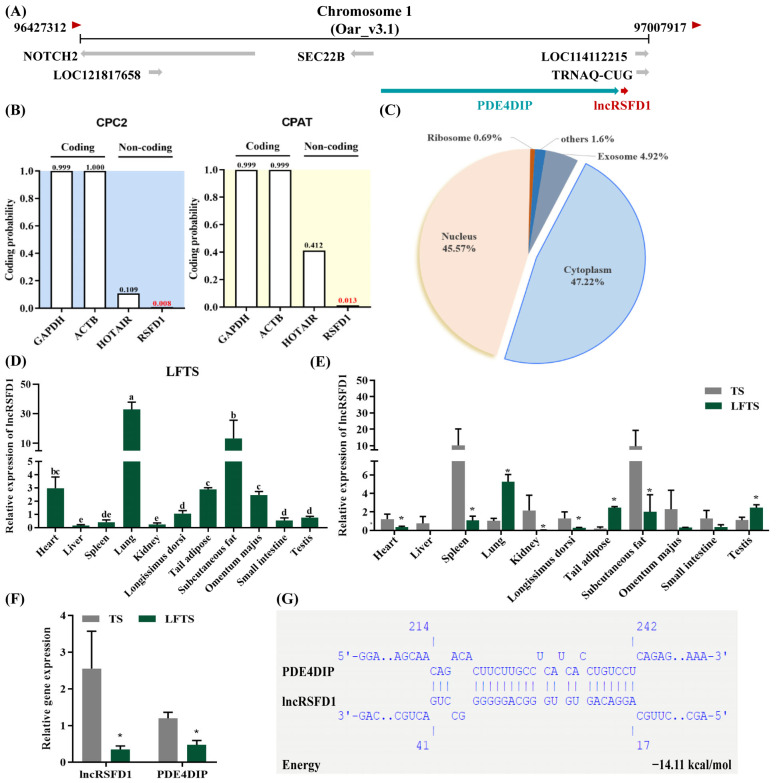
Molecular characteristics, tissue expression, and its regulated neighboring genes of ovine *lncRSFD1*. (**A**) Genome location; (**B**) evaluating the protein coding capacity of *lncRSFD1* through CPC2 and CPAT; (**C**) subcellular location (lncLocator 1.0); (**D**) different tissues mRNA expression pattern of *lncRSFD1* in Lanzhou fat-tailed sheep (LFT); (**E**) comparison of the expression of *lncRSFD1* in different tissues of LFT and Tibetan (TS) sheep; (**F**) expression of *lncRSFD1* and PDE4DIP in the tail adipose of different sheep breeds; (**G**) the interaction between lncRSFD1 and PDE4DIP mRNA 3′UTR was predicted by IntaR-NA2.0. * *p* < 0.05, and different lowercase letters or indicated significant (*p* < 0.05) differences.

A 490-nt lncRNA sequence (TCONS_00054953) from previous RNA-seq was named *lncRSFD1* for its potential role in regulating fat deposition in sheep [4]. Located on sheep chromosome 1 downstream of the *PDE4DIP* gene, *lncRSFD1* is a forward transcribed intergenic lncRNA (Figure 1A). ORF Finder, CPC2, and CPAT analyses confirmed it lacks significant coding potential, similar to the known lncRNA HOTAIR (Figure 1B). The secondary structure of lncRNA, crucial for lncRNA-protein interactions, exhibits conserved features. RNAfold predictions showed that *lncRSFD1* has a significant presence of single-stranded and loop structures, with a minimum free energy of −147.40 kcal/mol (Figure S1A–C). lncLocator 1.0 predicts *lncRSFD1* localization in both the cytoplasm and nucleus (Figure 1C). Although no orthologous gene was found in humans, mice, and bovines, downstream regions of the *PDE4DIP* gene in humans and mice contain lncRNAs (ENSG00000254539 and ENSMUSG00000086557) that may be homologous to *lncRSFD1* (Figure S1D–E). *lncRSFD1* was expressed in various tissues of different sheep breeds. In LFT sheep, it was highest in the lungs, and significantly higher in subcutaneous and tail adipose tissue than in the liver, spleen, and kidney (Figure 1D). Compared to TS sheep, LFT sheep had higher expression in the lung, testis, and tail adipose tissue (Figure 1E).

### 3.2. The Proliferation Inhibition and Differentiation Promotion of Preadipocytes by lncRSFD1

It is hypothesized that *lncRSFD1* may affect the proliferation and differentiation of precursor adipocytes, since it is highly expressed in adipose tissue, so the functional experiments were conducted using the 3T3-L1 murine preadipocyte cell.

#### 3.2.1. The Effects of Overexpressing *lncRSFD1* on 3T3-L1 Cell Proliferation

**Figure 2:**
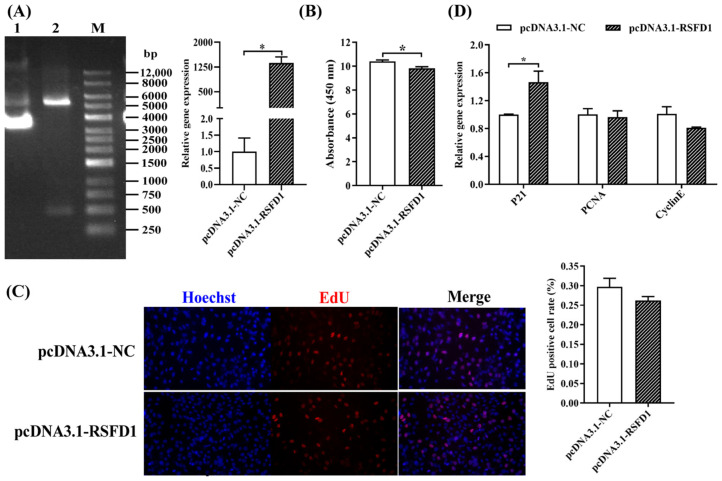
Overexpression of *lncRSFD1* inhibited the proliferation of preadipocytes. (**A**) The construction and detection of *lncRSFD1* overexpression vector; the effect of the *lncRSFD1* overexpression on 3T3-L1 cell proliferation was detected by (**B**) CCK-8 and (**C**) EdU (magnification: 200×); (**D**) the expression of mRNA of cell proliferation genes. Note: (left A) Double digestion of pcDNA3.1-RSFD1. lane 1: pcDNA3.1 (+); lane 2: Plasmid digested with *HindIII*-*EcoRI*; lane M: DNA Marker. (Right A) Overexpression efficiency of the *lncRSFD1* in 3T3-L1 cells (* *p* < 0.05).

According to the results of the overexpression efficiency, the *lncRSFD1* overexpression vector was available (Figure 2A). The results of CCK-8 showed that *lncRSFD1* overexpression significantly inhibited 3T3-L1 cell proliferation (Figure 2B), and EDU staining confirmed a lower proliferation index in the overexpression group compared to the control (Figure 2C). Overexpression of *lncRSFD1* down-regulated *PCNA* and *CyclinE*, while significantly up-regulating the anti-proliferation marker P21 (Figure 2D), indicating that *lncRSFD1* inhibits 3T3-L1 preadipocyte proliferation.

#### 3.2.2. Effect of *lncRSFD1* Overexpression on the Differentiation of 3T3-L1 Cells

**Figure 3:**
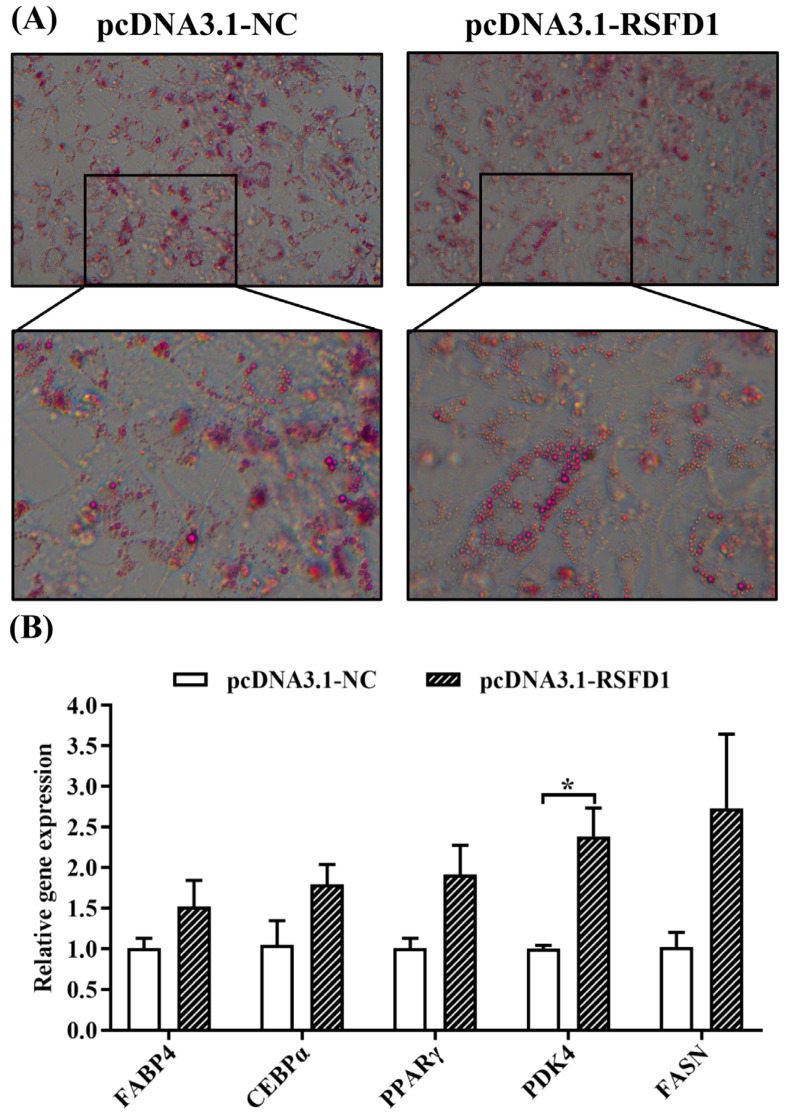
Effect of *lncRSFD1* on differentiation of 3T3-L1 preadipocytes. (**A**) Oil red O staining of 3T3-L1 cells (magnification: 100×); (**B**) the mRNA expression of adipogenic differentiation-related genes (* *p* < 0.05).

Oil red O staining was performed on 3T3-L1 cells at 5 days of differentiation, and the results showed that compared with the control group, the preadipocytes in the *lncRSFD1* overexpression group had obvious circular lipid droplet accumulation around the nucleus (Figure 3A). After overexpression of *lncRSFD1*, the mRNA expression levels of *PDK4* were significantly higher than those in the control (Figure 3B). These results indicate that overexpression of *lncRSFD1* promotes the differentiation of 3T3-L1 cells.

#### 3.2.3. Predication of Potential Molecules Targeted by *lncRSFD1*

lncRNAs regulate the expression of protein-coding genes at their synthesis site or multiple adjacent genes through cis-acting elements located on the flanking region of protein-coding genes [20]. Co-expression analysis of differentially expressed mRNAs and *lncRSFD1* based on previous RNA-seq data [4] revealed that *lncRSFD1* interacts with 67 genes, including *MYH1*, *PFKM*, *PYGM*, *CACNA1S*, *PPP1R3A*, *TCAP*, and *PDE4DIP* (Figure S1F). The differential expression of *PDE4DIP* within 10 kb upstream of *lncRSFD1* was consistent with *lncRSFD1* in the tail-fat tissues of LFT sheep and TS (Figure 1F). IntaRNA 2.0 predicted an interaction between *lncRSFD1* and *PDE4DIP* mRNA 3′UTR, supported by high sequence similarity (98.57%) (Figure 1F).

**Figure 4:**
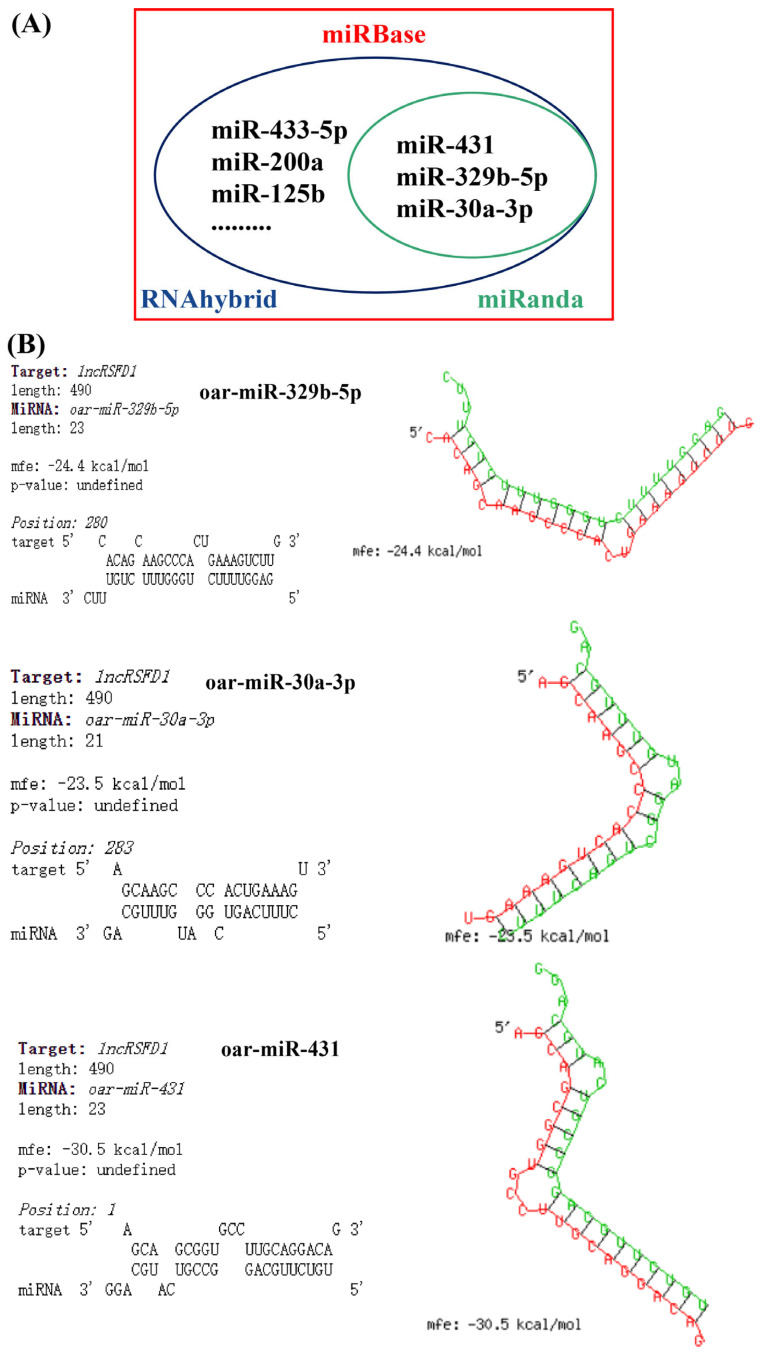
Prediction of miRNAs adsorbed by *lncRSFD1* (**A**,**B**) the secondary structure of *lncRSFD1*-miRNA pairs.

The potential target relationships between *lncRSFD1* and 153 mature sheep miRNA sequences, including *oar-miR-30a-3p*, *oar-miR-329b-5p*, and *oar-miR-431*, were predicted using miRanda and RNAhybrid (Figure 4A). It was indicated that *lncRSFD1* may be potentially targeted by the above three miRNAs (Figure 4B). Among them, *oar-miR-30a-3p* exhibits high conservation across multiple species with identical seed sequences (UUUCAGU), while *oar-miR-329b-5p* shares homology with miRNAs from other species such as common cattle (Table S2). Additionally, *miR-431* shows conservation across several species with an identical seed sequence (GUCUUGCA) (Table S2).

### 3.3. Identification of lncRSFD1 Core Promoter Region

**Figure 5:**
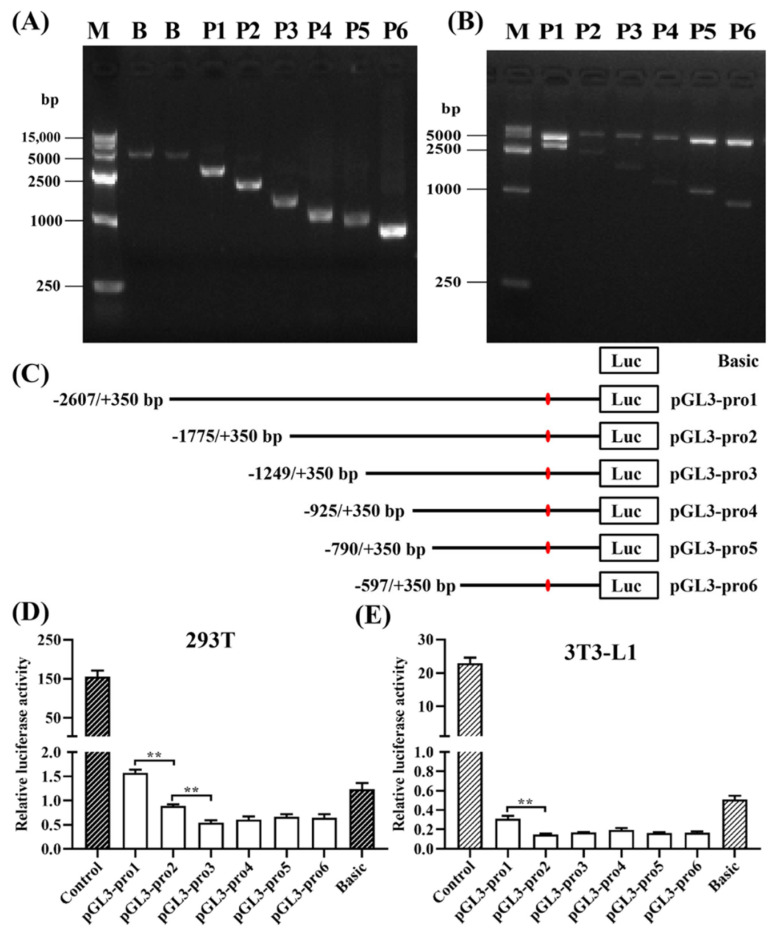
Identification of the core promoter region of *lncRSFD1*. (**A**) The PCR amplification of *lncRSFD1* promoter deletion fragments and (**B**) double enzyme digestion for recombinant plasmid vectors, and (**C**) schematic diagram of deletion vector and (**D**,**E**) the luciferase reporter activity detection of sheep *lncRSFD1* promoter reconstructed plasmids (** *p* < 0.01).

According to predictions from BDGP and Softberry, six potential promoter regions (score > 0.80) were identified within the *lncRSFD1* −3000 bp to +500 bp region (Table S3). By using a method of fixed 3′ flanking region and gradually shortened 5′ end, six promoter sequences of *lncRSFD1* with decreasing lengths were obtained, producing bands of 2957, 2125, 1599, 1275, 1140, and 947 bp, as confirmed by agarose gel electrophoresis (Figure 5A,C). These fragments were ligated to the pGL3-Basic vector, and sequencing confirmed the recombinant plasmids. Electrophoresis analysis after double enzyme digestion with KpnI and XhoI showed expected results, indicating the successful construction of six promoter series deletion luciferase reporter vectors: pGL3-pro1 (−2607 bp to +350 bp), pGL3-pro2 (−1775 bp to +350 bp), pGL3-pro3 (−1249 bp to +350 bp), pGL3-pro4 (−925 bp to +350 bp), pGL3-pro5 (−790 bp to +350 bp), and pGL3-pro6 (−597 bp to +350 bp) (Figure 5A,B). The dual-luciferase reporter assay revealed the core promoter was located between −2607 bp and −1776 bp (Figure 5D,E).

### 3.4. Identification of SNPs in the Core Promoter Region of lncRSFD1

**Figure 6:**
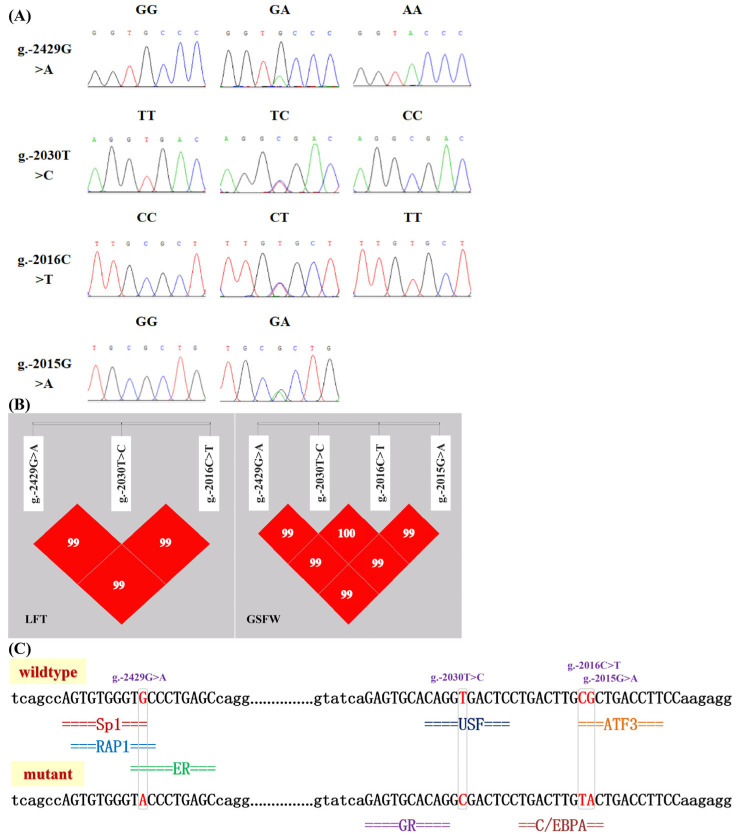
Identification of genetic variants in the core promoter region of *lncRSFD1*. (**A**) Sequencing peak of SNPs sites in sheep *lncRSFD1* core promoter; (**B**) Linkage disequilibrium plot of the SNP sites in sheep *lncRSFD1* core promoter; (**C**) Prediction of transcription factors potentially binding to the sequence containing SNPs in the core promoter region of sheep *lncRSFD1*. Note: Lanzhou fat-tailed sheep (LFT) and Guiqian semi-fine wool sheep (GSFW).

DNA sequencing identified four SNPs in the core promoter region of *lncRSFD1* in LFT and GSFW: g.-2429G>A, g.-2030T>C, g.-2016C>T, and g.-2015G>A (Figure 6A). The GG genotype of the g.-2429G>A locus was dominant in both populations, with frequencies of 0.519 in LFT and 0.826 in GSFW. The dominant allele at this locus was G (Table 1).

In both LFTS and GSFW populations, the g.-2429G>A locus has three genotypes (GG, GA, AA), with GG being dominant (frequencies of 0.519 and 0.826, respectively), and the g.-2030T>C and g.-2016C>T loci have three genotypes (TT, TC, CT), with the heterozygous mutant genotype being dominant. The g.-2015G>A locus is monomorphic in the LFTS population (GG genotype only) and has both GG (dominant) and GA genotypes in the GSFW population (Table 1). The χ^2^ goodness-of-fit test indicates all four SNP loci are in Hardy–Weinberg equilibrium (HWE) in the LFTS population, while g.-2030T>C and g.-2016C>T deviate from HWE in the GSFW population. The g.-2030T>C and g.-2016C>T loci have relatively rich genetic diversity and are moderately polymorphic, whereas g.-2429G>A and g.-2015G>A in the GSFW population show lower variability and were categorized as low polymorphic (Table 2).

Moreover, in the LFTS population, except for the monomorphic g.-2015G>A locus, the g.-2429G>A, g.-2030T>C, and g.-2016C>T loci were in strong linkage disequilibrium (D′ = 1, r^2^ = 0.754), with g.-2030T>C and g.-2016C>T being completely linked (D′ = 1, r^2^ = 1, Figure 6B). In the GSFW, all four loci showed strong linkage disequilibrium (D′ > 0.99), with g.-2030T>C and g.-2016C>T still completely linked (D′ = 1, Figure 6B).

### 3.5. Association Analysis of Promoter Subregion Polymorphism with Ovine Traits

Genotypic correction excluded genotypes with a frequency lower than 5%, and an analysis of the association between remaining genotypes and eight growth traits, such as body weight, was conducted. Due to fewer than 10 effective individuals per genotype in the LFS population when divided by gender, the association analysis was only performed in the GSFW population.

The g.-2429G>A locus had no significant impact on GSFW body measurements (*p* > 0.05), and due to complete linkage disequilibrium between g.-2030T>C and g.-2016C>T loci, only the association analysis results of the g.-2030T>C locus with sheep growth traits were presented. In the ram GSFW, the g.-2030T>C mutation was significantly associated with body height in rams, with heterozygous individuals exhibiting significantly greater body height compared to the wild-type and mutant individuals (*p* < 0.05) (Table 3). Similarly, the g.-2030T>C mutation was significantly associated with the body weight, body height, body length, chest depth, and cannon circumference in GSFW ewes, and all these traits showed that the growth trait indicators of mutant individuals were highly significant compared to wild-type individuals (*p* < 0.01) (Table 3).

Compared to the genetic effects of individual mutation sites on complex traits, the analysis of multiple linked genetic variations, forming haplotypes and diplotypes, is more practically significant in evaluating their influence on traits.

In GSFW, these 4 SNP loci collectively formed 5 haplotypes (H1′ to H5′) and 7 diplotypes, with H1′ being the most frequent haplotype (GTCG, 0.533). Among diplotypes, H1′H2′ was dominant (GG-TC-CT-GG, frequency: 0.351), while H1′H3′ (GA-TT-CC-GG) and H3′H3′ (AA-TT-CC-GG) were recessive (frequency: 0.011 each, Table 4). Analysis showed that individuals with H1′H3′ diplotypes had lower body height and weight compared to others (*p* < 0.05, Table 5). In ewes, H1′H3′ individuals had lower body weight (*p* < 0.05). Additionally, H1′H1′ and H1′H3′ individuals had lower body height than H2′H2′ (*p* < 0.01), while H3′H3′ individuals had shorter body length than H2′H2′ (*p* < 0.05). Chest circumference of H1′H1′ and H1′H4′ individuals was higher than H1′H3′, H2′H2′, and H3′H3′ (*p* < 0.05), and girth of H2′H2′ individuals was greater than H1′H5′ and H3′H3′ (*p* < 0.05) (Table 5).

The g.-2429G>A mutation prevents the binding of transcription factors (TFs) SP1 (specificity protein 1), RAP1 (RAS-associated protein 1), and ER (estrogen receptors) to *lncRSFD1*’s core promoter. The g.-2030T>C mutation changes binding TFs from upstream stimulatory factor (USF) to glucocorticoid receptor (GR). The adjacent mutations g.-2016C>T and g.-2015G>A cause the disappearance of the binding site for the transcription factor *ATF3* (activating transcription factor 3) (Figure 6C). Different lowercase superscript letters (a, b and c) indicate 0.01 < *p* < 0.05, while different uppercase superscript letters (A and B) indicate *p* < 0.01. a, b, and c, 0.01 < *p* < 0.05.

## 4. Discussion

*LncRSFD1*, differentially expressed in the tail fat of sheep, was found to be expressed across various sheep tissues, with higher levels in the subcutaneous fat, tail fat, and greater omentum of LFT and TS sheep. Notably, the tissue expression patterns of *lncRSFD1* were different between the two sheep breeds, suggesting breed-specific regulatory roles. This study further revealed that *lncRSFD1* inhibits the proliferation of preadipocytes while promoting their differentiation. Cell proliferation and differentiation are two fundamental, intricately coordinated processes impacting fat deposition in multicellular organisms [29]. In this study, *lncRSFD1* primarily inhibits fat cell proliferation by promoting the expression of the cell cycle inhibitor *P21*. Regarding the regulation of 3T3-L1 cell differentiation, overexpression of *lncRSFD1* upregulates the mRNA expression of genes related to lipogenesis, such as *FASN* and *PPARγ*, as well as the key lipid metabolism gene *PDK4*. PDK4 primarily regulates fat metabolism and glucose metabolism by affecting processes such as glycolysis, the tricarboxylic acid cycle, and ATP production [30]. In goat intramuscular fat cells, PDK4 promotes lipid accumulation by regulating the expression of genes related to triglyceride synthesis and lipid deposition, including *CD36*, *FABP3*, *ACACA*, and *AGRAT6* [31]. Additionally, *PDK4* and *perilipin 2* genes are differentially expressed in the tail fat of Tibetan sheep and LFS [4]. Thus, it is speculated that *lncRSFD1* regulates 3T3-L1 cell differentiation by modulating the lipid metabolism pathway.

The function of lncRNAs is related to their subcellular localization, and *lncRSFD1* is distributed in both the cytoplasm and the nucleus, suggesting that it may exert its functions through multiple molecular pathways. lncRNAs can regulate the expression of adjacent genes in a cis-regulatory manner [32]. For example, LOC100847835 can influence fat development by regulating the nearby *C*/*EBPβ* gene [33]. This study initially analyzed the protein-coding genes within a 10 kb region upstream and downstream of *lncRSFD1* and identified the *PDE4DIP* gene, also known as *myomegalin* (*MMGL*) or *cardiomyopathy-associated 2* (*CMYA2*) gene. Previous research has suggested that *PDE4DIP* is a candidate key gene involved in fat deposition regulation [34]. In this study, the expression of *lncRSFD1* and the *PDE4DIP* showed a consistent differential trend in the tail fat of LFS and Tibetan sheep. Bioinformatic predictions indicated a potential interaction between *lncRSFD1* and the 3′UTR of *PDE4DIP* mRNA. Therefore, it is speculated that one mechanism by which *lncRSFD1* influences adipose growth and development is by regulating the expression of the *PDE1DIP* gene.

Furthermore, considering the expression of *lncRSFD1* in the cytoplasm and additional target predictions, it was found that *lncRSFD1* may sponge adsorb *oar-miR-30a-3p*, *oar-miR-329b-5p*, and *oar-miR-431*. These miRNAs are highly conserved between species and play roles in both fat- and muscle-related physiological processes. For instance, *oar-miR-30a-3p* has significantly higher expression in the tail fat of Bashbay sheep (fat-tailed type) compared to second-generation crossbred sheep of wild Argali sheep and Bashbay sheep (thin-tailed type) [35]. *miR-30a-3p* interacts with *PPAR*α to alleviate triglyceride accumulation and liver adipose deposition [36]. *miR-431* primarily regulates processes such as cancer development and progression, muscle development, and regeneration [37]. Additionally, *miR-431* can target the *IRS2* gene to inhibit adipogenic differentiation of human bone marrow mesenchymal stem cells (hMSCs) [38]. Based on this, it is suggested that another mechanism of action of *lncRSFD1* is to regulate the differentiation of precursor adipose cells by targeting *oar-miR-30a-3p*, *oar-miR-329b-5p*, and *oar-miR-431*. However, the regulatory relationships between *lncRSFD1* and miRNAs, as well as between *lncRSFD1* and the *PDE4DIP* gene, still require experimental validation to clarify the specific molecular mechanisms. For example, luciferase reporter assays could be used to confirm the direct interaction between *lncRSFD1* and the 3′UTR of *PDE4DIP* mRNA, while RNA immunoprecipitation (RIP) assays could validate the miRNA sponging role of *lncRSFD1*. Additionally, loss- and gain-of-function experiments in primary sheep adipose cells could provide insights into how *lncRSFD1* affects the differentiation of precursor adipose cells and lipid metabolism. Furthermore, investigating the function of *lncRSFD1* in regulating precursor adipose cell differentiation and lipid metabolism in primary sheep adipose cells is essential for future research. Future work should also focus on elucidating the upstream regulators and downstream effectors of *lncRSFD1* to build a more comprehensive understanding of its role in adipose growth and development.

In most cases, lncRNAs may not have specific functions, but their transcription is essential, and the promoter of lncRNAs exhibits considerable conservation, similar to protein-coding genes [39,40]. The core promoter region of *lncRSFD1* was identified to be between −2607 bp and −1776 bp from the transcription start site. Genetic variations in promoter regions can influence gene transcription activity, and mutations in these regions can lead to phenotypic changes in livestock [41,42]. Currently, several genetic markers associated with important economic traits have been identified in the promoter regions of livestock lncRNAs. For instance, a 12-bp InDel (rs720343880) variation in the promoter region of the adipose tissue-specific lncRNA LOC100847835 is significantly associated with cattle body characteristics [33]. In this study, four SNP sites in the core promoter region of *lncRSFD1* were further identified and analyzed.

Livestock growth traits and body traits are moderately heritable (0.20~0.35), and the identification of genetic markers associated with these traits can significantly enhance molecular marker-assisted selection (MAS) breeding and gene editing breeding, thereby shortening the breeding cycle and reducing breeding costs [43,44]. This study analyzed the association of four SNP sites with sheep growth traits and found that the polymorphism and haplotypes of the four candidate sites were significantly associated with the weight, body length, and chest width of GSFW. This suggests that *lncRSFD1* may be a potential functional molecule related to sheep growth traits and meat production traits. SNPs in gene promoter regions can regulate gene transcription activity by changing the affinity of TFs to their binding sites. For example, a 19-bp repeat sequence in the promoter of the lincRNA NORFA enhances the transcription of lincRNA NORFA by recruiting more TF NFIX to its promoter region, which is involved in follicular atresia and granulosa cell apoptosis, thereby regulating the reproductive capacity of sows [45]. Online predictions suggest that SNPs in the core promoter region of *lncRSFD1* affect the binding of several transcription factors. Specifically, mutations in this region reduce the binding affinity of SP1, USF, and ATF3 while allowing GR and C/EBPα to bind to the mutated sequence. Transcription factors such as ATF3, SP1, GR, and C/EBPα are known to regulate genes involved in lipid metabolism [46,47,48]. However, further research is necessary to understand the impact of SNPs in the *lncRSFD1* promoter region on *lncRSFD1* transcriptional activity and the molecular mechanisms by which SNPs affect sheep growth traits.

In summary, this study identified a novel non-coding RNA, *lncRSFD1*, which suppresses proliferation and promotes differentiation of precursor adipose cells. Polymorphisms in its promoter region were significantly associated with sheep growth traits. These findings provide new scientific insights into the biological functions and regulatory mechanisms of ncRNAs in sheep adipose tissue and offer potential candidate sites for the application of MAS to accelerate sheep breeding.

## 5. Conclusions

This study identified a novel non-coding RNA, lncRSFD1lncRSFD1, which plays a dual role in suppressing proliferation and promoting differentiation of precursor adipose cells. Furthermore, polymorphisms in its promoter region showed significant associations with sheep growth traits. These findings enhance our understanding of the biological roles and regulatory mechanisms of ncRNAs in sheep adipose tissue and highlight potential candidate markers for MAS to improve sheep breeding efficiency.

## Figures and Tables

**Table 1:** Genotype and allele frequency of SNPs in the ovine *lncRSFD1* core promoter.

Loci	Breeds	Number	Genotype Frequencies			Allele Frequencies	
			Wildtype	Heterozygote	Homozygous Mutant	Wildtype	Mutant
g.-2429G>A			GG	GA	AA	G	A
	LFT	52	0.519 (27)	0.442 (23)	0.039 (2)	0.740	0.260
	GSFW	350	0.826 (289)	0.163 (57)	0.011 (4)	0.907	0.093
g.-2030T>C			TT	TC	CC	T	C
	LFT	52	0.058 (3)	0.519 (27)	0.423 (22)	0.317	0.683
	GSFW	350	0.403 (141)	0.406 (142)	0.191 (67)	0.606	0.394
g.-2016C>T			CC	CT	TT	C	T
	LFT	52	0.058 (3)	0.519 (27)	0.423 (22)	0.317	0.683
	GSFW	350	0.403 (141)	0.406 (142)	0.191 (67)	0.606	0.394
g.-2015G>A			GG	GA	AA	G	A
	LFT	52	1.000 (52)	0	0	1.000	0
	GSFW	350	0.986 (345)	0.014 (5)	0	0.993	0.007

Note: LFT, Lanzhou fat-tailed sheep; GSFW, Guiqian semi-fine wool sheep. The same is below.

**Table 2:** Genetic parameters of SNPs in the ovine *lncRSFD1* core promoter.

Loci	Breeds	Number	Ho	Ne	PIC	HWE *p*-Value
g.-2429G>A	LFT	52	0.616	1.625	0.311	*p* > 0.05
	GSFW	350	0.832	1.203	0.154	*p* > 0.05
g.-2030T>C	LFT	52	0.567	1.764	0.339	*p* > 0.05
	GSFW	350	0.522	1.914	0.364	*p* < 0.05
g.-2016C>T	LFT	52	0.567	1.764	0.339	*p* > 0.05
	GSFW	350	0.522	1.914	0.364	*p* < 0.05
g.-2015G>A	LFT	52	1.000	1.000	0	*p* > 0.05
	GSFW	350	0.986	1.014	0.014	*p* > 0.05

Note: Ho, homozygosity; Ne, effective number of alleles; PIC, polymorphic formation content; HWE, Hardy–Weinberg equilibrium.

**Table 3:** The significant correlation between g.-2030T>C and growth traits of GSFW.

	Genotypes (Mean ± SE)			*p*-Value
	Wildtype	Heterozygous Genotype	Homozygous Mutant	
Ram	TT (*n* = 12)	TC (*n* = 32)	CC (*n* = 22)	
body height (cm)	69.08 ^b^ ± 1.09	72.47 ^a^ ± 0.85	69.95 ^b^ ± 0.73	0.027
Ewe	TT (*n* = 129)	TC (*n* = 110)	CC (*n* = 45)	
body weight (kg)	43.41 ^B^ ± 0.69	46.10 ^A^ ± 0.79	48.30 ^A^ ± 1.13	0.001
body height (cm)	64.09 ^C^ ± 0.50	66.60 ^B^ ± 0.56	70.24 ^A^ ± 0.72	3.33 × 10^−9^
body length (cm)	68.51 ^C^ ± 0.65	72.05 ^B^ ± 0.87	76.67 ^A^ ± 1.09	1.63 × 10^−8^
chest depth (cm)	32.99 ^B^ ± 0.31	33.80 ^AB^ ± 0.34	34.99 ^A^ ± 0.62	0.006
cannon circumference (cm)	8.33 ^B^ ± 0.07	8.65 ^A^ ± 0.07	8.83 ^A^ ± 0.07	4.9 × 10^−5^

Note: Values with different lowercase letters or uppercase letters within the same row indicated significant (*p* < 0.05) or extremely significant differences (*p* < 0.01).

**Table 4:** Haplotype and diplotype analysis of *lncRSFD1* core promoter SNP sites in LFT and GSFW sheep.

Breeds	Name	Types	Number	g.-2429G>A	g.-2030T>C	g.-2016C>T	g.-2015G>A	Frequency
LFT	Haplotypes	H1	28	G	T	C	-	0.269
		H2	49	G	C	T	-	0.471
		H3	5	A	T	C	-	0.048
		H4	22	A	C	T	-	0.212
	Diplotypes	H1H2	5	GG	TC	CT	-	0.096
		H1H3	1	GA	TT	CC	-	0.019
		H1H4	22	GA	TC	CT	-	0.423
		H2H2	22	GG	CC	TT	-	0.423
		H3H3	2	AA	TT	CC	-	0.038
Guiqian semi-fine wool sheep	Haplotypes	H1′	371	G	T	C	G	0.533
		H2′	256	G	C	T	G	0.368
		H3′	46	A	T	C	G	0.066
		H4′	19	A	C	T	G	0.027
		H5′	4	G	T	C	A	0.006
	Diplotypes	H1′H1′	94	GG	TT	CC	GG	0.270
		H1′H2′	122	GG	TC	CT	GG	0.351
		H1′H3′	38	GA	TT	CC	GG	0.109
		H1′H4′	19	GA	TC	CT	GG	0.055
		H1′H5′	4	GG	TT	CC	GA	0.011
		H2′H2′	67	GG	CC	TT	GG	0.193
		H3′H3′	4	AA	TT	CC	GG	0.011

**Table 5:** Association of diplotypes with growth traits of GSFW sheep.

Gender/Traits	Diplotypes (Mean ± SE)							*p*-Value
	H1′H1′	H1′H2′	H1′H3′	H1′H4′	H1′H5′	H2′H2′	H3′H3′	
**Ram number**	*n* = 8	*n* = 26	*n* = 3	*n* = 5	-	*n* = 22	-	
body weight (kg)	48.40 ± 3.32	46.13 ± 1.12	39.38 ± 1.91	49.48 ± 2.41	-	43.60 ± 1.35	-	0.090
**body height (cm)**	**70.25 ^a^ ± 1.28**	**72.23 ^a^ ± 1.01**	**65.33 ^b^ ± 1.20**	**72.80 ^a^ ± 1.46**	**-**	**69.95 ^a^ ± 0.73**	**-**	**0.049**
body length (cm)	73.75 ± 2.30	73.77 ± 0.96	71.00 ± 0.58	76.60 ± 2.32	-	72.95 ± 0.98	-	0.550
chest depth (cm)	36.75 ± 1.16	35.65 ± 0.65	35.33 ± 1.45	36.80 ± 0.66	-	35.27 ± 0.73	-	0.765
chest width (cm)	25.63 ± 1.00	26.73 ± 0.58	25.33 ± 0.88	27.20 ± 0.58	-	26.14 ± 0.53	-	0.690
chest circumference (cm)	89.75 ± 2.48	89.00 ± 0.98	82.00 ± 1.15	92.60 ± 2.32	-	87.86 ± 1.30	-	0.129
cannon circumference (cm)	10.00 ± 0.19	10.23 ± 0.12	10.00 ± 0.00	10.00 ± 0.00	-	10.00 ± 0.09	-	0.512
Ewe number	*n* = 86	*n* = 96	*n* = 35	*n* = 14	*n* = 4	*n* = 45	*n* = 4	
**body weight (kg)**	**43.98 ^b^ ± 0.86**	**45.62 ^ab^ ± 0.74**	**42.01b ^c^ ± 1.24**	**49.36 ^a^ ± 3.57**	**43.60 ^b^ ± 4.97**	**48.30 ^a^ ± 1.13**	**43.25 ^b^ ± 3.84**	**0.019**
**body height (cm)**	**64.01 ^B^ ± 0.61**	**66.55 ^AB^ ± 0.59**	**64.06 ^B^ ± 1.00**	**66.93 ^AB^ ± 1.71**	**64.25 ^AB^ ± 2.95**	**70.24 ^A^ ± 0.72**	**65.75 ^AB^ ± 1.89**	**6.61 × 10^−7^**
**body length (cm)**	**68.36 ^ab^ ± 0.80**	**71.66 ^ab^ ± 0.91**	**68.91 ^ab^ ± 1.32**	**74.71 ^ab^ ± 2.79**	**69.75 ^ab^ ± 3.84**	**76.67 ^a^ ± 1.09**	**67.00 ^b^ ± 1.22**	**0.023**
chest depth (cm)	33.24 ± 0.38	33.73 ± 0.36	32.44 ± 0.63	34.29 ± 0.97	33.00 ± 1.58	34.99 ± 0.62	32.50 ± 1.19	0.067
chest width (cm)	27.09 ± 0.31	27.38 ± 0.28	26.74 ± 0.53	28.71 ± 1.12	26.50 ± 1.26	28.09 ± 0.43	26.50 ± 0.65	0.191
**chest circumference (cm)**	**99.32 ^a^ ± 0.95**	**97.49 ^ab^ ± 0.82**	**94.54 ^b^ ± 1.47**	**102.07 ^a^ ± 2.55**	**98.75 ^ab^ ± 2.02**	**96.11 ^b^ ± 1.00**	**95.00 ^b^ ± 3.14**	**0.024**
**cannon circumference (cm)**	**8.31 ^abc^ ± 0.08**	**8.64 ^ab^ ± 0.07**	**8.43 ^abc^ ± 0.12**	**8.68 ^ab^ ± 0.25**	**8.00 ^bc^ ± 0.71**	**8.83 ^a^ ± 0.07**	**7.88 ^c^ ± 0.04**	**0.010**

## Data Availability

Data are available upon request from corresponding authors.

## Supplementary Materials

The following supporting information can be downloaded at: https://www.mdpi.com/article/10.3390/ani14243631/s1, Supplemental Figure S1. The secondary structure, expression and network of lncRSFD. Supplemental Table S1. Information regarding the primers used. Supplemental Table S2. Mature sequences of miRNAs adsorbed by *lncRSFD1* in different species. Supplemental Table S3. Prediction of the promoter region of *lncRSFD1* using BDGP and Softberry.
