# Supplementary material for: Ovine LncRSFD1 Mined from RNA-Seq: Identification, Expression Profile, Promotion of Preadipocyte Differentiation, Promoter Activity, and Its Polymorphisms Related to Phenotypic Traits
Source: Animals (Basel). 2024 Dec 16;14(24):3631. doi: 10.3390/ani14243631 (PMC11672851; doi:10.3390/ani14243631)
Supplement: Supplementary file 1 [file animals-14-03631-s001.zip › animals-3307456-supplementary.pdf]

Supplemental Figures：

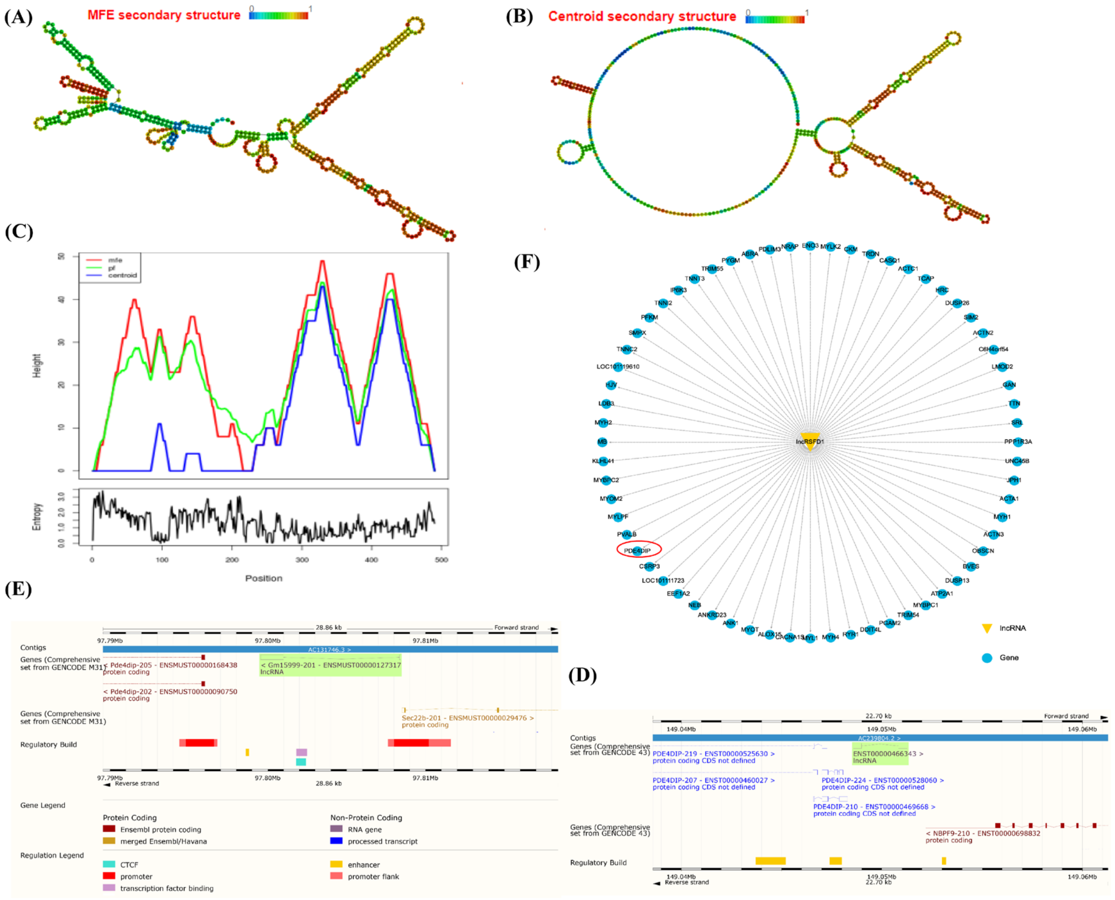

Supplemental Figure 1. The secondary structure, expression, and network of lncRSFD. (A) Minimum free energy (MFE) and (B) centroid secondary structure (CEN) prediction result. (C) Thermodynamics peak diagram of the *lncRSFD1* MFE structure; lncRNA located in the 3′ downstream of the PDE4DIP gene in (D) human and (E) mouse genomes. (F) The co-expression networks of *lncRSFD1* and differentially expressed genes (plotted from published data, Ma et al. 2018).

Supplemental Table 1 Information regarding the primers used.

Primers Names	Primer sequences (5' to 3')	Size (bp)	Reference sequences	Application
oar-lncRSFD1	F: GCCTAGTCTGTGTCTTCACAAAAG<br>R: CAGTTATGTTAGCCTTCCACACAG	112	*lncRSFD1*	mRNA expression profile
oar-PDE4DIP	F: AGCGGAAAGAACGGATGCTG<br>R: AGCAGGCCCTGAATCTCCAT	85	XM_027973716.2	
oar-GAPDH	F: CCTGCCAAGTATGATGAGAT<br>R: TGAGTGTCGCTGTTGAAGT	119	NM_001190390.1	
mmu-PPARγ	F: GTGCCAGTTTCGATCCGTAGA<br>R: GGCCAGCATCGTGTAGATGA	142	NM_011146.4	
mmu-FABP4	F: CAGAAGTGGGATGGAAAGTCG<br>R: CGACTGACTATTGTAGTGTTTGA	169	NM_024406.4	
mmu-C/EBPα	F: CAAGAACAGCAACGAGTACCG<br>R: GTCACTGGTCAACTCCAGCAC	124	NM_001287514.1	
mmu-PDK4	F: AAACCCAAGCCACATTGGAAGTA<br>R: CGCAGAGCATCTTTGCACAC	89	NM_013743.2	
mmu-FASN	F: ATTGGCTCCACCAAATCCAAC<br>R: CCCATGCTCCAGGGATAACAG	90	NM_007988.3	qRT-PCR
mmu-CyclinE	F: CCGTCTTGAATTGGGGCAATA<br>R: GAGCTTATAGACTTCGCACACC	167	NM_007633.2	
mmu-PCNA	F: GAACCTCACCAGCATGTCCA<br>R: ATTCACCCGACGGCATCTTT	221	NM_011045.2	
mmu-P21	F: CCTGGTGATGTCCGACCTG<br>R: CCATGAGCGCATCGCAATC	103	NM_007669.5	
mmu-β-actin	F: GGCTGTATTCCCCTCCATCG<br>R: CCAGTTGGTAACAATGCCATGT	154	NM_007393.5	
			**REases**	
Pro-R	CCGCTCGAGGGGCCTCTTGCATGTCACTTA		*XhoI*	
Pro-F1	CGGGGTACCGACCACTGACAGAGCACCTATT	2957		luciferase reporter vector construction
Pro-F2	CGGGGTACCGGTCTGCCTCTGAATGAGCTT	2125		
Pro-F3	CGGGGTACCTCTGATTCCTTTCTTTCCTGAGCC	1599	*KpnI*	
Pro-F4	CGGGGTACCCCCCATCTCCTCATCAAGTAAACA	1275		
Pro-F5	CGGGGTACCACAAAAGCCATCCTCTTTCTTCCA	1140		
Pro-F6	CGGGGTACCCTTGACAGGCTCTCACCACAA	947		

Note, REases means restriction endonuclease. The underlined letter indicates the restriction site.

Supplemental Table2 Mature sequences of miRNAs adsorbed by *lncRSFD1* in different species.

miRNAs	Names	Species	Mature Sequences（5′→3′）
miR-30a-3p	oar-miR-30a-3p	Ovis aries	C**UUUCAGU**CGGAUGUUUGCAG
	chi-miR-30a-3p	Capra hircus	C**UUUCAGU**CGGAUGUUUGCAG
	gga-miR-30a-3p	Gallus gallus	C**UUUCAGU**CGGAUGUUUGCAGC
	ssc-miR-30a-3p	Sus scrofa	C**UUUCAGU**CGGAUGUUUGCAGC
	hsa-miR-30a-3p	Homo sapiens	C**UUUCAGU**CGGAUGUUUGCAGC
	mml-miR-30a-3p	Macaca mulatta	C**UUUCAGU**CGGAUGUUUGCAGC
	ggo-miR-30a-3p	Gorilla gorilla	C**UUUCAGU**CGGAUGUUUGCAGC
	mmu-miR-30a-3p	Mus musculus	C**UUUCAGU**CGGAUGUUUGCAGC
	rno-miR-30a-3p	Rattus norvegicus	C**UUUCAGU**CGGAUGUUUGCAGC
miR-329	oar-miR-329b-5p	Ovis aries	G**AGGUUUUC**UGGGUUUCUGUUUC
	bta-miR-329b	Bos taurus	A**GAGGUUUU**CUGGGUUUCUGUUU
	pal-miR-329b-5p	Pteropus alecto	A**GAGGUUUU**CUGGGUUUCUGUUU
	cfa-miR-329a	Canis familiaris	A**GAGGUUUU**CUGGGUUUCUGUUU
miR-431	oar-miR-431	Ovis aries	U**GUCUUGCA**GGCCGUCAUGCAGG
	bta-miR-431	Bos taurus	U**GUCUUGCA**GGCCGUCAUGCAGG
	hsa-miR-431-5p	Homo sapiens	U**GUCUUGCA**GGCCGUCAUGCA
	mml-miR-431	Macaca mulatta	U**GUCUUGCA**GGCCGUCAUGCA
	mmu-miR-431-5p	Mus musculus	U**GUCUUGCA**GGCCGUCAUGCA
	rno-miR-431	Rattus norvegicus	U**GUCUUGCA**GGCCGUCAUGCA
	cpo-miR-431-5p	Cavia porcellus	U**GUCUUGCA**GGCCGUCAUGCA

Supplemental Table 3 Prediction of the promoter region of *lncRSFD1* using BDGP and Softberry.

Software	Start	End	Score	Promoter Sequence
BDGP	425	475	0.91	AGGGAGAGTCTATGAAAAAGGAGCTTCTGGAACTGAGAAC**C**AAACTATCC
	1291	1341	0.99	TATGCCACTATATAAAAAACTGATGGAAAGATGCCACCTA**A**CAAGTTGAT
	2156	2206	0.82	GGGCCACTTCTATATGTGCCTGTTTCAAGCTAAGCATATC**T**TTTCCTGGG
	2593	2643	0.94	ACCGGAGGTCCTTAAAACAGCAGGAAGGATGGGCCTGTGC**C**CCTTCTTCA
	2699	2749	0.85	TGGAAGGGGTCATAGATATGGCCTCGTGCGCAGGGTGAAA**G**GCAGAGGGC
	2753	2803	0.83	CCTGCACTTGAGGAATAACAAGGCAGGGTGCCCGTGGCTC**A**GCTCTAAGG
	3430	3480	0.89	GATATATATTTATATATGTTGCTATAGTGCTGTGTGGAAG**G**CTAACATAA
	**Position**	**LDF**		**TATA box**
Soft-berry	2191	+1.997		2166 +4.820 TATATGTG
	2633	+0.373		2603 +4.049 CTTAAAAC
	1331	-0.513		1299 +7.629 TATATAAA
